# Maintaining Optimal Surgical Conditions With Low Insufflation Pressures is Possible With Deep Neuromuscular Blockade During Laparoscopic Colorectal Surgery: A Prospective, Randomized, Double-Blind, Parallel-Group Clinical Trial

**DOI:** 10.1097/MD.0000000000002920

**Published:** 2016-03-07

**Authors:** Myoung Hwa Kim, Ki Young Lee, Kang-Young Lee, Byung-Soh Min, Young Chul Yoo

**Affiliations:** From the Department of Anesthesiology and Pain Medicine (MHK, KYL, YCY); Anesthesia and Pain Research Institute (MHK, KYL,YCY); and Division of Colon and Rectal Surgery, Department of Surgery (KYL, BSM), Yonsei University College of Medicine, Seodaemun-gu, Seoul, Republic of Korea.

## Abstract

Carbon dioxide (CO_2_) absorption and increased intra-abdominal pressure can adversely affect perioperative physiology and postoperative recovery. Deep muscle relaxation is known to improve the surgical conditions during laparoscopic surgery. We aimed to compare the effects of deep and moderate neuromuscular block in laparoscopic colorectal surgery, including intra-abdominal pressure.

In this prospective, double-blind, parallel-group trial, 72 adult patients undergoing laparoscopic colorectal surgery were randomized using an online randomization generator to achieve either moderate (1–2 train-of-four response, n = 36) or deep (1–2 post-tetanic count, n = 36) neuromuscular block by receiving a continuous infusion of rocuronium. Adjusted intra-abdominal pressure, which was titrated by a surgeon with maintaining the operative field during pneumoperitoneum, was recorded at 5-minute intervals. Perioperative hemodynamic parameters and postoperative outcomes were assessed.

Six patients from the deep and 5 from the moderate neuromuscular block group were excluded, leaving 61 for analysis. The average adjusted IAP was lower in the deep compared to the moderate neuromuscular block group (9.3 vs 12 mm Hg, *P* < 0.001). The postoperative pain scores (*P* < 0.001) and incidence of postoperative shoulder tip pain were lower, whereas gas passing time (*P* = 0.002) and sips of water time (*P* = 0.005) were shorter in the deep neuromuscular block than in the moderate neuromuscular block group.

Deep neuromuscular blocking showed several benefits compared to conventional moderate neuromuscular block, including a greater intra-abdominal pressure lowering effect, whereas surgical conditions are maintained, less severe postoperative pain and faster bowel function recovery.

## INTRODUCTION

Compared with open surgery, laparoscopic surgery has the advantages of reduced intensity of postoperative pain and hospital stay, and improved cosmetic results and patient satisfaction.^1^ Laparoscopic surgery generally involves insufflation of carbon dioxide (CO_2_) into the peritoneal cavity producing a pneumoperitoneum; this causes an increase in the intra-abdominal pressure (IAP). However, CO_2_ absorption and elevated IAP during pneumoperitoneum can cause specific pathophysiological effects such as cardiovascular, pulmonary, and splanchnic perfusion changes.^2^ Thus far, several studies have been conducted in an effort to reduce CO_2_ pressure and minimize adverse effects of pneumoperitoneum and have reported postoperative pain relief after low-pressure pneumoperitoneum.^3–5^ However, in these studies, an adequate surgical field or conditions were not considered.

In previous studies, deep muscle relaxation was shown to improve surgical conditions in laparoscopic cholecystectomy, urologic, and gynecologic surgery,^6–8^ and the risk of a delayed respiratory function recovery due to a deep neuromuscular block (NMB) was avoided by using sugammadex.^9,10^ Sugammadex (Bridion, Merck Sharp and Dohme – MSD, Oss, the Netherlands), a selective relaxant binding agent, is a modified g-cyclodextrin, especially created to bind the free plasma molecules of the neuromuscular blocking agent rocuronium to which it has high affinity.^11^ However, there is little evidence of a deep NMB effect that would allow the clinical use of a lower insufflation pressure in laparoscopic surgery.

In the present study, we aimed to evaluate the extent of IAP reduction of a deep NMB against a conventional moderate NMB, while maintaining a satisfactory surgical exposure, and compare the perioperative physiological functions and postoperative recovery profiles of patients who received deep or moderate NMB during laparoscopic colorectal surgery.

## METHODS

This was a single-center, prospective, double-blind, randomized controlled trial. The study protocol was approved by the Institutional Review Board and Hospital Research Ethics Committee of Severance Hospital at the Yonsei University Health System in Seoul, Republic of Korea, on 21 July on 2014 (# 4–2014–0559). This was registered at clinicalTrials.gov (NCT02266056) on 26 August on 2014. The informed consent was given to the all subjects, and written consent was obtained from all patients. Adult patients, who underwent elective laparoscopic colorectal resection surgery and had the American Society of Anesthesiologists (ASA) physical status classification of I–III, were assessed for eligibility. Exclusion criteria included: (1) patients aged >80 years, (2) inability to provide informed consent, (3) allergy to rocuronium or sugammadex, (4) a neuromuscular disease or personal history or family history of malignant hyperthermia, (5) a serious heart, kidney, or liver condition, (6) previous colorectal surgical history and obesity (body mass index ≥ 35 kg/m^2^).

Patients, who were enrolled in this clinical trial, were randomly assigned to the deep NMB or moderate NMB group in a 1:1 ratio by using a computer-generated random number table (available at www.randomizer.org/form.htm), and assignments were concealed in a sealed envelope. The randomization was not blocked or stratified.

On arrival in the operating room, routine monitoring was started and it included electrocardiography, pulse oximetry, noninvasive blood pressure, and capnography. Anesthetic depth was monitored using a bispectral index (BIS) monitor (Aspect A-2000^®^, Aspect Medical system Inc, Newton, MA). Patients were given 0.2 mg of glycopyrrolate intravenously (IV), and anesthesia was induced with a bolus administration of 1.5 to 2 mg/kg of propofol and 1 to 2 mg/kg of remifentanil. Anesthesia was maintained using 4 to 7% desflurane with an adjuvant IV infusion of 0.05 to 0.2 μg/kg/min of remifentanil. Rocuronium, which is a neuromuscular relaxing agent, 0.6 mg/kg was injected to facilitate tracheal intubation in all patients. Tracheal intubation was performed in female and male patients using a 6.5-mm and 7.5-mm (internal diameter) tracheal tube, respectively. The cuff pressure of the tracheal tube was maintained at 20 to 25 cm H_2_O throughout the procedure. Mechanical ventilation was maintained with a tidal volume of 8 mL/kg, and the ventilatory frequency was adjusted to maintain an end-tidal CO_2_ concentration of 35 to 45 mm Hg with an air/oxygen mixture (fraction of inspired oxygen 0.5). Body temperature was maintained at 36 °C to 37°C. BIS scores were maintained in the range of 40 and 60, and mean arterial pressure within 20% of preinduction values. After intubation, arterial cannulation was performed to monitor intraoperative continuous arterial blood pressure and perform arterial blood gas analysis during surgery.

After induction, rocuronium was continuously infused and titrated according to the group assignment, to maintain either the post-tetanic count (PTC) at 1 to 2 in the deep NMB group, or train-of-four (TOF) response at 1 to 2 in the moderate NMB group.^12^ The monitoring of quantitative neuromuscular transmission with a TOF-Watch or TOF SX device was applied to all patients. Neuromuscular function using an acceleromyograph was measured at the wrist. The TOF-watch (or TOF SX) generates an electrical stimulus to the ulnar nerve and measures contractions adductor pollicis muscle^13^ (causing adduction of the thumb) through a sensor attached to the tip of the thumb, which was placed in a flexible adaptor that applied a constant preload to the thumb, using a current of 50 to 60 mA with a pulse width of 0.02 ms. This muscle relaxation power was maintained until completing closure of the abdominal fascia. The surgeons, patients, and those assessing outcomes were blinded to the group assignment.

At 30 minutes before the end of the surgery, ramosetron 0.3 mg IV for prophylaxis of nausea and vomiting, and paracetamol 1 g for analgesia were administered. Intravenous patient-controlled analgesia (PCA) consisting of fentanyl 15 μg/kg and nefopam 120 mg mixed with normal saline to comprise a total volume of 100 mL was administered at a basal rate of 2 mL/hour, with a bolus dose of 0.5 mL and a 15-minute lockout time, for postoperative pain control in all patients. Upon completion of the surgery, sugammadex 4 or 2 mg/kg IV was used to reverse any possible residual neuromuscular blocking effects in both groups. The tracheal tube was removed when a TOF ratio of 0.9 and spontaneous ventilation were observed and then the patients were transferred to the post-anesthetic care unit (PACU). Patients were transferred to the ward after a minimum of 30 minutes following PACU admission and when they met the modified Aldrete scoring system discharge criteria (score ≥ 9 with no score of 1 in any individual category).^14^

Titrated average IAP during pneumoperitoneum was the primary endpoint. Setting IAP was recorded at 5-minute intervals. In both groups, the same CO_2_ pressure was initially applied for a pneumoperitoneum at 12 mm Hg, which was routine CO_2_ pressure in our institution for laparoscopic colorectal surgery, to expand the space within the abdominal cavity. Then, the IAP was controlled as lowly as possible by a surgeon with maintaining the same operative field compared with that of initial IAP 12 mm Hg. The surgeon continuously titrated the IAP to create a necessary operating space.

Secondary outcomes were (1) overall surgical conditions, including visible field and muscle relaxation, estimated by the surgeons using the 5-stage satisfaction (extremely poor/ poor/ acceptable/ good/ optimal), (2) perioperative respiratory and cardiovascular parameters measured and documented at the following time points: before the induction, before the incision, 1 hour after the pneumoperitoneum, and 10 minutes after the end of the pneumoperitoneum, (3) postoperative recovery profiles, assessed by an blinded investigator, including: postoperative abdominal pain intensity, the incidence of shoulder tip pain, analgesic consumption, antiemetic requirement, bowel function recovery parameters (gas passing time, sips of water [SOW] time, and soft diet time), and hospital stay, (4) pain and nausea intensity evaluated using a numeric rating scale (NRS) with a 0 (no pain or nausea) to 10 (worst pain imaginable or nausea) scoring system at 4 time periods (during PACU stay, 1–6 hours, 6–24 hours, and 24–48 hours after surgery).

### Statistical Analysis

Because there were no previous studies that compared to quantify the difference of IAPs between the patients who received deep NMB and those received moderate NMB, sample size was not calculated. Instead, to analyze the data with normal distribution, 30 patients in each group would be required at least. Therefore we considered in a 20% dropout rate and enrolled 36 patients in each group.

Comparisons were made on an intention-to-treat basis, as it is evident from the results that only the patients who actually received allocated intervention were analyzed. A 2-sided *P* value of <0.05 was considered statistically significant for all comparisons. Continuous variables are summarized as mean ± standard deviation (SD) or median (interquartile range [IQR]) as appropriate, whereas nominal variables are summarized as number of subjects (proportion, %). Normality was tested using the Kolmogorov–Smirnov test as appropriate. Between-group comparisons of continuous variables were performed with Student's 2 sample *t* test or the Mann–Whitney *U* test as appropriate. Nominal and categorical variables were compared with the Chi-square test and Fisher's exact test. All analyses were conducted using IBM SPSS 21.0 (IBM Corp., Armonk, NY).

## RESULTS

Seventy-two patients, who underwent laparoscopic colorectal resection surgery between August 2014 and August 2015, were enrolled in this study. Five patients in the deep NMB group and 3 patients in the moderate NMB group dropped out after randomization due to conversions to open colorectal resection or concurrent other operations. In addition, 1 patient in the deep NMB group and 2 patients in the moderate NMB group were lost to follow-up during hospital stay because of refusal of patients. The remaining 61 patients were finally analyzed. Table 1 shows baseline demographic and clinical patient characteristics; there were no significant differences between the 2 groups. There were no harms or unintended effects in both groups.

**TABLE 1:**
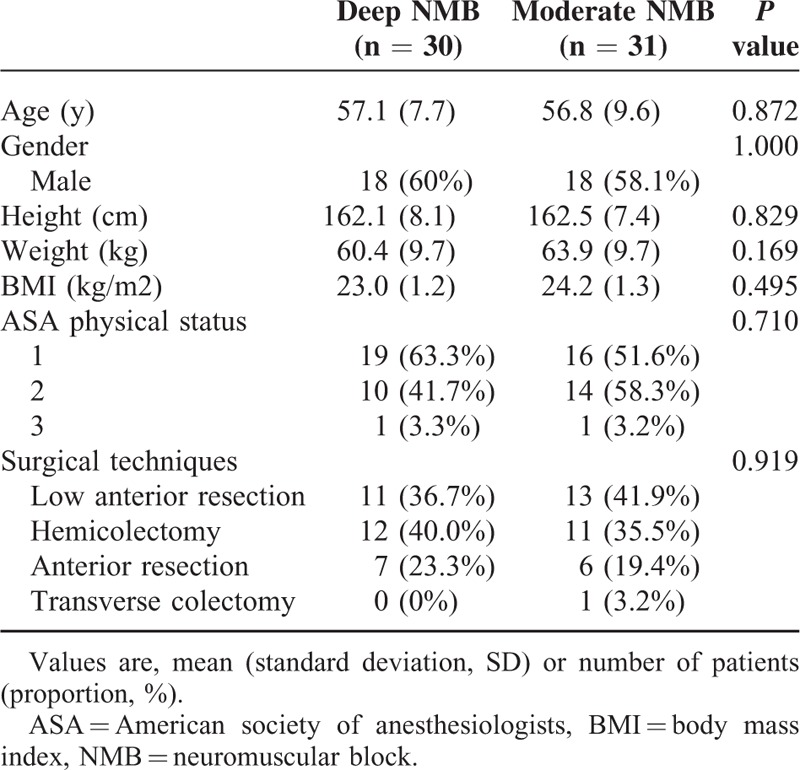
Baseline Demographic and Clinical Patient Characteristics

### Intra-abdominal Pressure and Other Perioperative Parameters

The titrated average IAP was significantly lower in the deep NMB group than in the moderate NMB group (9.3 ± 1.3 vs 12.0 ± 0.5 mm Hg, *P* < 0.001). The surgical condition was significantly superior in the deep NMB group compared with the moderate NMB group (*P* < 0.001). Intraoperative estimated blood loss (20.2 ± 13.7 vs 60.0 ± 95.3 mL, *P* = 0.029) and peak airway pressure after CO_2_ insufflation and deflation (21.6 ± 3.3 vs 25.0 ± 3.5 mm Hg, *P* < 0.001; 15.7 ± 2.1 vs 17.0 ± 2.9 mm Hg, *P* = 0.039, respectively) were significantly lower in the deep than in the moderate NMB group. A significantly greater total amount of rocuronium and sugammadex was needed among patients in the deep than in the moderate NMB group (Table 2).

**TABLE 2:**
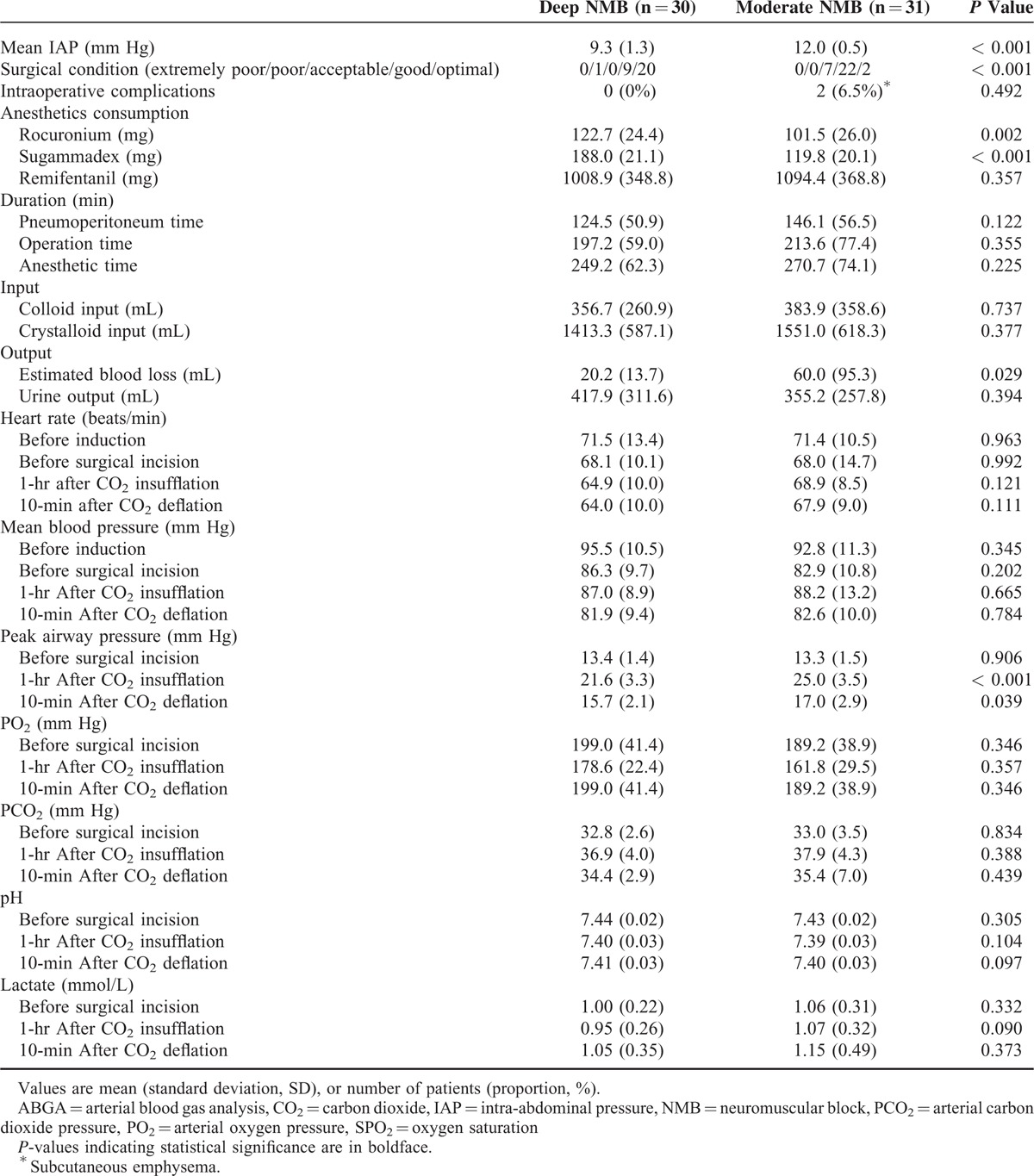
Perioperative Data

### Postoperative Recovery Profiles

The NRS pain scores were significantly lower in the deep than in the moderate NMB group at the PACU and at 6, 24, and 48 hours postoperatively (*P* < 0.001). The incidence of postoperative shoulder tip pain was also significantly lower in the deep than in the moderate NMB group (1 patient vs 8 patients, *P* = 0.026). Significantly lower amounts of IV morphine equivalents were administered within 6 to 24 hours after surgery in the deep than in the moderate NMB group (median 0.0, IQR 7.0 mg vs median 6.0, IQR 12.0 mg, *P* *=* 0.005). The number of patients who required additional rescue analgesics at 1 to 6, 6 to 24, and 24 to 48 hours after surgery was significantly lower in the deep than in the moderate NMB group (n = 13, 43% vs n = 25, 80.6%, *P* = 0.004; n = 9, 30 % vs n = 23, 74.2 %, *P* = 0.001; n = 9, 30% vs n = 19, 61.3%, *P* = 0.021, respectively). Regarding the bowel movement recovery time, gas passing time (median 40, IQR 11.3 vs median 64, IQR 31 hours, *P* = 0.002) and SOW time (median 16, IQR 5.4 vs median 19, IQR 6 hours, *P* = 0.005) were shorter in the deep than in the moderate NMB group (Table 3).

**TABLE 3:**
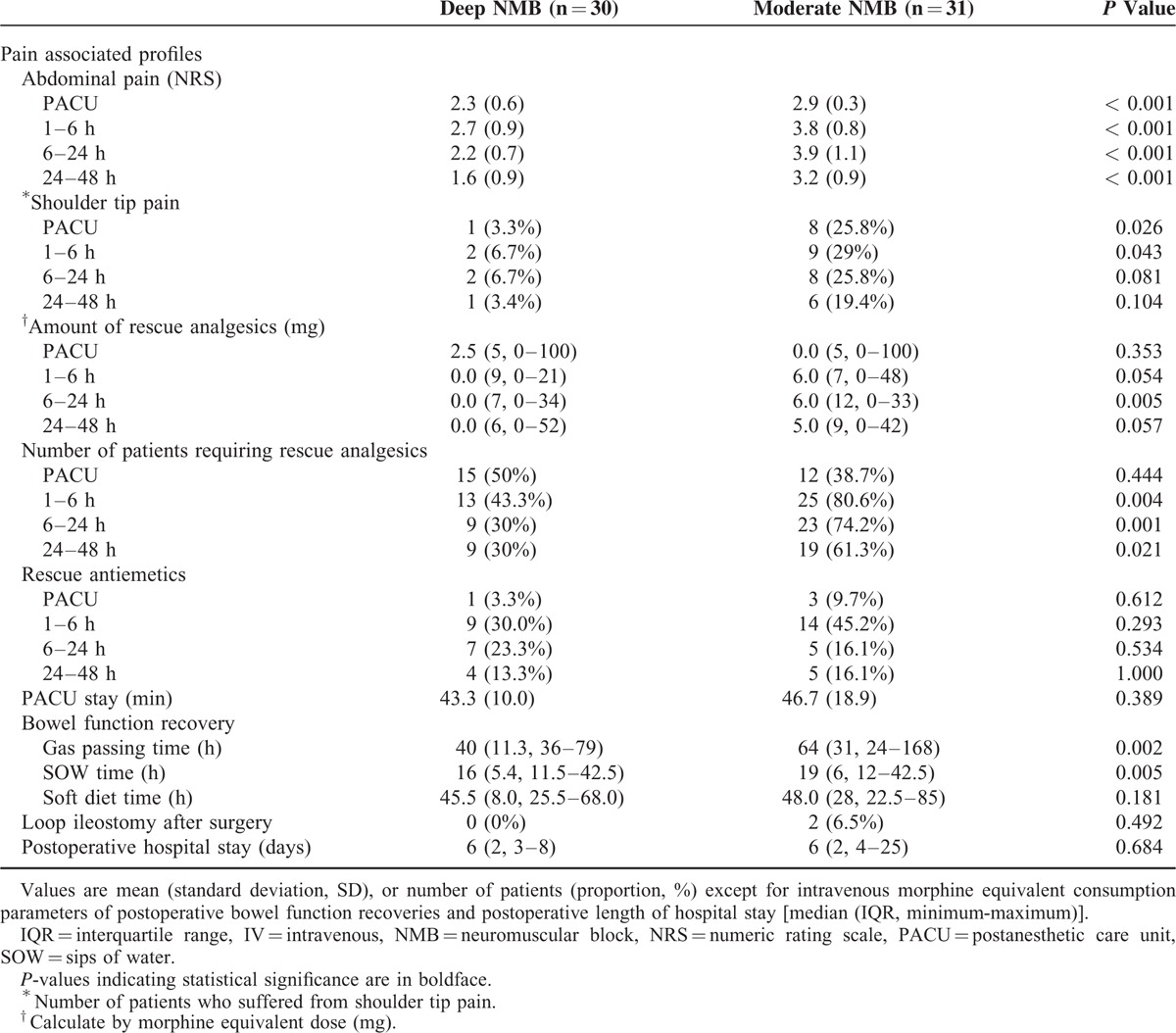
Patient Postoperative Recovery Profiles

## DISCUSSION

Increased IAP induced by the CO_2_ pneumoperitoneum has been thought to have negative effects on the intra-abdominal organs and cardiovascular and pulmonary systems.^2,15^ One international guideline recommended to “use the lowest intra-abdominal pressure allowing adequate exposure of the operative field, rather than using a routine pressure.”^1^ However, the authors of a recent systematic review concluded that “the recommendation to use low pressure pneumoperitoneum during laparoscopy is weak.”^16^ The most important benefit of low-pressure pneumoperitoneum reported in the studies included in this review was a lower intensity of postoperative pain, especially shoulder tip pain, and the influence of low IAP on the surgical field was not investigated in any of the studies.

Though some studies reported of an improved quality of surgical conditions by the use of deep rather than moderate NMB during retroperitoneal laparoscopy and laparoscopic cholecystectomy,^8,12^ the importance of pressure control during pneumoperitoneum remains controversial and subject to clinical research. In particular, no studies with major abdominal surgery settings, such as colorectal resection, have been conducted. The present study is the first clinical trial to show that laparoscopic colorectal surgery can be performed using the deep NMB and a lower CO_2_ insufflation pressure, without reducing surgical exposure. Our findings have shown that deep NMB produces a lower intra-abdominal CO_2_ insufflation pressure (9.3 mm Hg) compared to moderate NMB (12 mm Hg). Deep NMB has benefits of a lower intensity of postoperative pain and a faster bowel function recovery.

### Postoperative Pain and Recovery of Bowel Movement

Surgical pain after laparoscopic surgery is less severe and has a shorter duration than after open surgery^17^; it still causes considerable discomfort and an increased stress response.^18^ As the etiology of postlaparoscopic pain can be classified into at least 3 categories: visceral, incisional, and shoulder tip pain,^19–21^ we tried to assess different types of pain including abdominal pain and shoulder tip pain. In this trial, the patients in the deep NMB group reported a significantly lower intensity of postoperative abdominal pain at all periods within postoperative 48 hours and a lower intensity of shoulder tip pain within 6 hours postoperatively. The superior postoperative pain profiles in the deep NMB group are noteworthy findings of the current trial.

Generally, pain during the postoperative period could cause an activation of the inhibitory splanchnic reflexes and result in a postoperative ileus manifested by a delay in the first bowel motion, that leads to a delayed discharge after major abdominal surgery.^22^ In the present study, gas passing time and SOW time were significantly shorter in the deep versus the moderate NMB group, although hospital stay was similar between the groups. Schwarte et al^23^ found that an increasing IAP decreased gastric mucosal oxygen saturation. The European Association for Endoscopic Surgery guidelines^1^ also suggested that an elevated IAP mechanically compresses the capillary beds, decreases splanchnic microcirculation, and thus impairs oxygen delivery to the intra-abdominal organs. During pneumoperitoneum, a 24% blood flow reduction in the superior mesenteric artery and the hepatic portal vein was reported.^24^

Laparoscopic colorectal resection surgery is a major surgery ^25^ with duration longer than any other laparoscopic surgery previously researched in similar settings. The surgical duration affects the intra-abdominal organ perfusion.^1,26^ Current favorable postoperative pain and bowel function recovery results might be associated with a preserved intra-abdominal organ perfusion.

### Intraoperative Surgical Condition, Physiologic Change, and Estimated Blood Loss

Achieving a satisfactory operative field was not an aim but rather a precondition in our study, more optimal surgical condition was reported by the surgeons regarding the overall surgical conditions in the deep NMB group. For the evaluation of overall surgical conditions, the surgeons were asked to consider their satisfaction with 5-stage, including the surgical field. The reason for this finding may be that the deep NMB seems to not only enhance the surgical view, but also prevent involuntary diaphragm movements and provide a more comfortable position for surgeons to close the abdominal muscles and fascia.^27^ In addition, it is possible that the inducement and maintenance of a deep NMB alleviates securing a satisfactory intraperitoneal working space despite a lower insufflation pressure.^28^

In the present study, the hemodynamic parameters and arterial blood gas analysis results did not differ significantly between the 2 groups. This finding is consistent with findings from previous studies.^29^ However, the peak airway pressures 1 hour after CO_2_ insufflation and 10 minutes after deflation were lower in the deep NMB group compared to the moderate NMB group. These findings suggest that deep muscle relaxation may be beneficial in patients with obesity and respiratory comorbidity.

The estimated intraoperative blood loss was lower in the deep compared to the moderate NMB group. There is no evidence that deep muscle relaxation or low IAP are associated with lesser intra-abdominal bleeding; therefore further research is needed on the association of muscle relaxation and estimated intraoperative blood loss.

### Limitations

This study has several limitations. First, we could not measure the total CO_2_ consumption during pneumoperitoneum, because of a central CO_2_ supply in our center. Second, the maintenance of a deep NMB has important economic repercussions. Namely, the price of sugammadex is higher than that of traditional reversing agents (pyridostigmine or neostigmine), which makes deep NMB less accessible for patients of lower socioeconomic status. Finally, the secondary endpoint analysis was underpowered, considering the low number of subjects. Further trials are needed to confirm the benefits of deep NMB over moderate NMB in laparoscopic colorectal resection.

## CONCLUSIONS

Our findings suggest that deep NMB has benefits over conventional moderate NMB in laparoscopic surgery, including a greater IAP lowering effect, whereas surgical conditions are maintained, less severe postoperative pain and faster bowel function recovery. Therefore, low-pressure pneumoperitoneum with deep NMB is worth considering for patients undergoing laparoscopic surgery.
